# Integrative approach using liver and duodenum RNA**-**Seq data identifies candidate genes and pathways associated with feed efficiency in pigs

**DOI:** 10.1038/s41598-017-19072-5

**Published:** 2018-01-11

**Authors:** Yuliaxis Ramayo-Caldas, Maria Ballester, Juan Pablo Sánchez, Olga González-Rodríguez, Manuel Revilla, Henry Reyer, Klaus Wimmers, David Torrallardona, Raquel Quintanilla

**Affiliations:** 1 Animal Breeding and Genetics Program, Institute for Research and Technology in Food and Agriculture (IRTA), Torre Marimon, Caldes de Montbui, 08140 Spain; 2 Animal Genomics Department, Centre for Research in Agricultural Genomics (CRAG), Campus UAB, Bellaterra, 08193 Spain; 3 Departament de Ciència Animal i dels Aliments, Universitat Autònoma de Barcelona (UAB), 08193 Bellaterra, Spain; 4 Institute of Genome Biology, Leibniz Institute for Farm Animal Biology (FBN), Wilhelm-Stahl-Allee 2, Dummerstorf, 18196 Germany; 5 Faculty of Agricultural and Environmental Sciences, University Rostock, Rostock, 18059 Germany; 6 Animal Nutrition Program, IRTA, Mas de Bover, Constantí, 43120 Spain

## Abstract

This study aims identifying candidate genes and pathways associated with feed efficiency (FE) in pigs. Liver and duodenum transcriptomes of 37 gilts showing high and low residual feed intake (RFI) were analysed by RNA-Seq. Gene expression data was explored through differential expression (DE) and weighted gene co-expression network analyses. DE analysis revealed 55 and 112 differentially regulated genes in liver and duodenum tissues, respectively. Clustering genes according to their connectivity resulted in 23 (liver) and 25 (duodenum) modules of genes with a co-expression pattern. Four modules, one in liver (with 444 co-expressed genes) and three in duodenum (gathering 37, 126 and 41 co-expressed genes), were significantly associated with FE indicators. Intra-module analyses revealed tissue-specific candidate genes; 12 of these genes were also identified as DE between individuals with high and low RFI. Pathways enriched by the list of genes showing DE and/or belonging to FE co-expressed modules included response to oxidative stress, inflammation, immune response, lipid metabolism and thermoregulation. Low overlapping between genes identified in duodenum and liver tissues was observed but heat shock proteins were associated to FE in both tissues. Our results suggest tissue-specific rather than common transcriptome regulatory processes associated with FE in pigs.

## Introduction

Feed efficiency (FE) has a major impact on profitability and consequently in economic sustainability of pig production, where feedstuffs represent the largest expenditure component, accounting for 60% to 70% of production costs^1,2^. Additionally, improving FE implies a reduction in the amount of minerals, heavy metals and greenhouse gases excreted per kg of meat produced^3^, thus reducing the environmental impact of pig production systems. These elements together with the need to optimize the use of feed resources make FE a major economically and socially important trait. Including FE in selection schemes has therefore/consequently become an emerging and challenging trend in pig breeding.

As a complex trait, FE is determined by both genetics and environmental factors. Heritability of feed utilization efficiency has ranged from 0.14 to 0.53 depending on the whether the trait measured was FE or residual feed intake (RFI)^4–6^. More recently, Berry and Crowley^7^ proposed a new FE trait, the residual intake and body weight gain (RIG), which additionally to RFI takes into account the difference between actual and predicted growth. Several authors reported phenotypic and genetic variation for RIG in cattle breeds^8–10^; to the best of our knowledge, there are no references to genetic parameters for RIG in pigs.

In the light of the existing genetic determinism for FE, several genome-wide association (GWAS) and differential expression (DE) analyses have been performed for the aforementioned FE phenotypes, and a number of polymorphisms and genes have been reported to be associated to either RFI, FCR or RIG in livestock, particularly in cattle^8,11–13^, poultry^14,15^ and pigs^16–18^. Both GWAS and DE analyses performed to date tend to apply multiple-testing across the whole genome/transcriptome but testing in isolation the association of, respectively, a single genetic variant or the expression level of a gene with FE phenotypes. The use of network-based approaches has emerged as an alternative tool to study complex traits. Weighted gene co-expression analyses (WGCNA) enable us to identify functionally-related modules of co-expressed genes and also to integrate gene-expression data with external information^19,20^. Moreover, by analysing modules of genes rather than a single gene it is often easier to predict their function, interactions as well as common regulatory mechanisms^21,22^.

The main goal of this study is to identify candidate genes and pathways associated with FE in pigs through both DE analysis and a network-based approach that combine liver and duodenum transcriptomic data with FE phenotypic information.

## Results and Discussion

### Feed efficiency phenotypes

The 37 females selected for transcriptome analyses were chosen among the most extreme animals regarding RFI. Analysed animals were therefore classified in two groups denoted hereafter as H_FE_ (high feed efficiency, corresponding to animals with low RFI) and L_FE_ (low feed efficiency, with high RFI). Phenotypic differences between these groups are shown in Table 1.

**Table 1:** Phenotypic information of analysed animals divergently classified in high and low feed efficiency (FE): mean (standard error) and significance of differences between groups for Residual feed intake (RFI), Feed conversion ratio (FCR), Residual intake and body weight gain (RIG), Average daily gain (ADG), Average daily feed intake (ADI), Metabolic weight at mid-point of the control period (MW) and Backfat thickness (BFT).

Phenotype	High FE		Low FE		p-value
RFI (kg feed)	−0.236	(0.030)	0.304	(0.031)	<0.0001*
FCR (kg feed/kg gain)	2.207	(0.043)	2.736	(0.044)	<0.0001*
RIG	1.801	(0.363)	−2.604	(0.372)	<0.0001*
ADG (kg/d)	0.938	(0.023)	0.994	(0.024)	0.0998
ADI (kg/d)	2.070	(0.053)	2.702	(0.055)	<0.0001*
MW (kg^0.75^)	21.673	(0.424)	22.550	(0.435)	0.1567
BFT (mm)	11.952	(0.519)	11.900	(0.532)	0.9442

As expected, the two groups diverging for FE differed significantly in all FE phenotypes, including the classification criteria RFI but also in FCR and RIG. RFI and FCR were significantly lower in most efficient animals (H_FE_) and showed a positive covariation between them (phenotypic correlation r = 0.95; p-value < 0.0001). Conversely, RIG showed an opposite pattern, with lower values in H_FE_ when compared to L_FE_ group. This result is consistent with the fact that RIG includes the residual gain plus the negative standardized RFI, so that positive RIG values whereas negative RFI are related to higher FE. Accordingly, a strong and negative phenotypic correlation between RIG and RFI was observed in our data (R = −0.93, p-value < 0.0001).

Regarding other production traits, selecting most extreme animals according to RFI culminates in most efficient females with much lower feed intake (630 g less of daily feed intake) compared to the less efficient group, which explained the increase in feed efficiency. The other production phenotypes did not show significant differences between H_FE_ and L_FE_ groups, but a tendency towards a lower growth rate (50 gr less of daily growth on average) that was not significant. These results agree well with those recently obtained by Reyer *et al*.^18^, who in a similar experiment selecting divergent animals according to RFI observed a significant decrease in daily feed intake of high-FE animals, but no differences between groups in growth and fat deposition.

### Mapping and annotation

A total of 2173.36 Millions of 75 bp paired-end reads (42.5 M–71.2 M per liver sample) and 2185.88 M of 75 bp paired-end reads (42.4 M–71.0 M per duodenum sample) were generated from liver and duodenum RNA-Seq experiments, respectively. Around 87% (84.3% to 89.4% in liver) and 83% (75.2% to 86.3% in duodenum) of reads were mapped to the reference pig genome Sscrofa10.2. The highest percentages of reads were mapped to exons (59–75% in liver, 52–69% in duodenum), while intergenic regions were in a range of 15–33% in liver tissue and 20–38% in duodenum tissue. The lowest percentage of reads was located in introns (6–10% in liver, 6–18% in duodenum).

### Genes and biological pathways identified by differential expression analysis between animals with high and low FE

An initial analysis to identify genes differentially expressed between L_FE_ (inefficient) and H_FE_ (efficient) animals was performed using edgeR software. According to the employed cut-off (FC > 1.5 and corrected p-value ≤ 0.05), 55 and 112 genes were identified as DE in liver and duodenum tissues, respectively (Supplementary Table S1), being approximately half of them (22 genes in liver and 75 genes in duodenum) up-regulated in the L_FE_ group in comparison to H_FE_. It should be noted that 20 of the identified DE genes, five DE in liver and 15 in duodenum, have been previously reported as DE in different tissues of pigs also divergently selected for RFI^23^ (Supplementary Table S2).

Four genes, two per tissue, were analysed by quantitative real-time PCR (qPCR) to validate the RNA-Seq results: *HSPH1* and *ATF3* genes in liver, and *CRYAB* and *HSPB8* genes in duodenum. When the pattern of gene expression levels was compared, strong correlations ranking from 0.65 to 0.99 between qPCR and RNA-Seq were observed, confirming a high reproducibility of the data. The highest correlation coefficient between RNA-Seq and qPCR expression levels corresponded to the liver tissue genes (r = 0.99 for *HSPH1* and r = 0.99 for *ATF3*), whereas moderate to high correlation values were obtained for duodenum tissue genes (r = 0.65 for *CRYAB* and r = 0.72 for *HSPB8)*. However, in agreement with the DE analysis performed with RNA-Seq data, significant gene expression differences between groups were confirmed using the RQ mean values (p-value < 0.05).

Only three genes (*RNF181, CNN1, GTSF1*) were commonly identified as DE in both liver and duodenum tissues. This is also consistent with results obtained in the aforementioned study^23^, in which a low overlapping of DE genes in liver, *longissimus* muscle and two adipose tissues of growing pigs was observed. *RNF181* participates in the degradation of muscle proteins through the ubiquitin-proteasome system^24^, and has been also described as DE in the skeletal muscle of animals with divergent lipid profiles, being overexpressed in the leaner pigs^25^. Next, to gain insight into the biological processes that differed between L_FE_ and H_FE_ groups in liver and duodenum tissues, the list of DE genes was explored using the core analysis included in Ingenuity Pathways Analysis (IPA). In agreement with the largest number of DE genes, duodenum samples showed more significantly enriched pathways than the liver (Supplementary Table S3). Despite the rare overlapping between the lists of DE genes identified in each tissue, 12 canonical pathways were significantly overrepresented in both liver and duodenum: LXR/RXR and FXR/RXR Activation, Production of Nitric Oxide and Reactive Oxygen Species in Macrophages, Acute Phase Response Signaling, Histidine Degradation VI, Bupropion Degradation and IL-12 Signaling and Production in Macrophages (Supplementary Table S3). These results point out the association of those biological pathways to FE in both liver and duodenum, but suggest a tissue-specific regulation of genes involved in these biological processes.

### Co-expressed gene modules and correlation with FE traits

**Figure 1:**
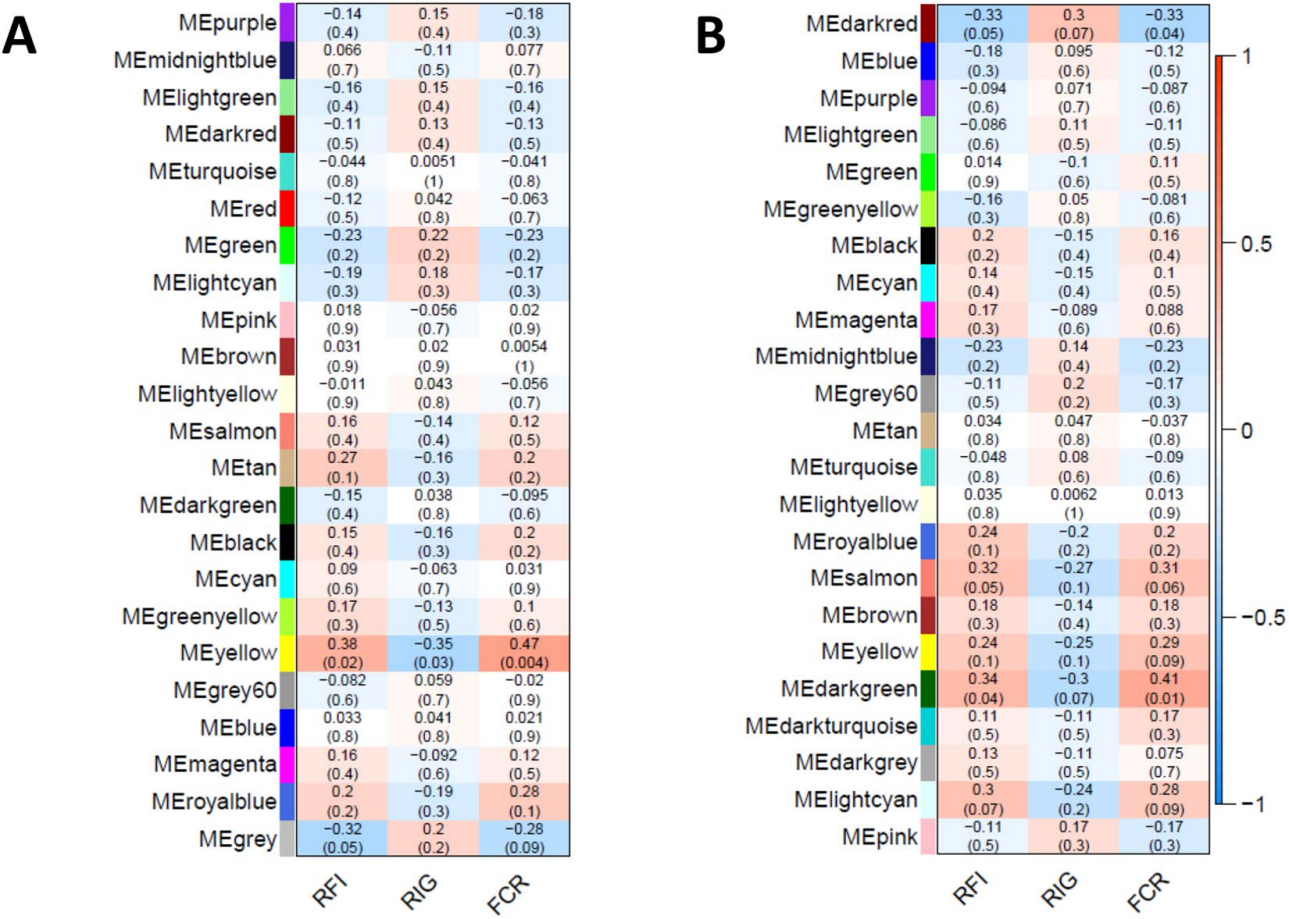
Gene co-expressed modules identified in liver (**A**) and duodenum (**B**), and correlation coefficients (p-values) of each module eigengene with residual feed intake (RFI), residual intake and body weight gain (RIG), and feed conversion ratio (FCR).

The covariation between gene-expression and phenotypic information, which usually is not considered in a classical DE analyses, could provide relevant information for disentangling the regulatory role of genes in a complex system. Therefore, in addition to DE analysis, we performed a WGCNA with gene expression data of liver and duodenum. Co-expression analyses revealed highly connected networks in both tissues. Genes were clustered according to their connectivity, and 23 and 25 modules of genes with a co-expression pattern were identified in liver and duodenum tissues, respectively. Supplementary table S4 provide the complete list of gene module membership in each tissue. WGCNA procedure assigned a specific colour to each gene-module that will be used to refer each module in the following sections. Figure 1 shows the colour assigned to each module, plus the correlation coefficient between the eigengene values of these modules and three FE phenotypes: RFI, FCR and RIG. In agreement with previous results^8^, opposite correlation patterns with the module eigengene values were observed between phenotype variation for RFI/FCR and RIG in both liver and duodenum tissues (Fig. 1). This is an expected result taking into account the negative relationship between RIG and RFI previously mentioned, so that positive RIG values whereas negative RFI are more favourable regarding FE.

The eigengene values of four of the identified gene modules, one in liver and three in duodenum, were significantly associated with RFI, FCR and RIG (Fig. 1). In liver, only the eigengene of Yellow module resulted to be significantly correlated with FE traits (Fig. 1A), reporting a positive correlation with RFI and FCR (r = 0.38 and 0.47, respectively), but negative correlation with RIG (r = −0.35). With 444 genes, the liver Yellow module contained the largest number of genes among the four gene modules significantly correlated to phenotypes. The same trend was observed in duodenum modules associated to FE (Fig. 1B; modules Salmon, Darkred and Darkgreen), being significantly correlated with FCR (all three modules) and RFI (except Salmon module) and contrarily correlated with RIG, albeit reaching statistical significance only at a suggestive level (p-values < 0.1). The Salmon and the Darkgreen modules included 126 and 37 co-expressed genes, respectively; both of them correlated positively with RFI and FCR, the correlation coefficients ranging from 0.31 to 0.41. Conversely, the duodenum Darkred module, that included 41 genes, was negatively correlated with RFI and FCR (r = −0.33 in both cases) and positively with RIG.

### Biological pathways deduced from gene modules associated with FE

Genes showing similar co-expression patterns (i.e. members of the same gene-module) either work cooperatively in related pathways and/or are under control of a common set of transcription factors (TF). Pathway analyses indicated that genes gathered in FE associated modules take part in a wide variety of physiological and biological events such as inflammation, immune response, lipid metabolism and thermoregulation (Supplementary Table S5). Among pathways significantly enriched it is worth highlighting Adipogenesis, Ephrin receptor signaling, EIF2 signaling, mTOR signaling, Phosphoinositide 3-kinase, ERK signalling, and Regulation of eIF4 and p70S6K signaling pathways, that had been previously reported as associated with FE in broilers, beef and pigs^11,18,23,26,27^. These pathways are essential for protein synthesis, and regulate many major cellular processes that generate or use large amounts of energy and nutrients.

When comparing across modules, eight pathways are overrepresented in at least two modules (Table 2). These pathways overrepresented in both tissues included Aldosterone Signaling in Epithelial Cells, NRF2-mediated Oxidative Stress Response and Unfolded protein response (liver-Yellow and duodenum-Darkgreen modules); Cell Cycle: G1/S Checkpoint Regulation, IL-1 Signaling, IL-8 Signaling (liver-Yellow and duodenum-Salmon modules); Role of RIG1-like Receptors in Antiviral Innate Immunity (liver-Yellow and duodenum-Darkred). Meanwhile, Protein Ubiquitination Pathway was common-overrepresented in the Yellow (liver), Darkgreen and Darkred (duodenum) modules. In spite of differences in the genetic background, age and diet of the animals included in our study compared to those analysed by Gondret *et al*.^23^, we identified genes belonging to pathways related to response to oxidative stress, protein metabolism, inflammation and immune response common in the two studies (Supplementary Table S5).

**Table 2:** Description of pathways overrepresented in co-expression modules associated to feed efficiency identified in both liver and duodenum tissues.

Cannonical Pathways	Tissues (Module)	-log(p-value)	Genes
Aldosterone Signaling in Epithelial Cells	duodenum (Darkgreen)	5.80	*HSPH1,DNAJB1,DNAJA1,HSPA2,HSPA4L*
	liver (Yellow)	4.79	*HSPA4,CRYAB,HSP90AB1,DNAJB4,SGK1,HSPH1,HSPB8,HSPD1,DNAJA1,HSPA2,DNAJB5,HSPA4L*
Cell Cycle: G1/S Checkpoint Regulation	duodenum (Salmon)	1.64	*RPL11,RPL5*
	liver (Yellow)	1.71	*MYC,SMAD3,CDKN2C,E2F3*
IL-1 Signaling	duodenum (Salmon)	1.35	*TRAF6,GNAS*
	liver (Yellow)	2.46	*FOS,JUN,NFKBIE,NFKB2,NFKB1,IRAK2*
IL-8 Signaling	duodenum (Salmon)	1.44	*TRAF6,GNAS,PGF*
	liver (Yellow)	4.72	*FOS,VCAM1,JUN,ICAM1,RND3,RHOB,HBEGF,VEGFC,LIMK2,PTGS2,NFKB1,FNBP1,IRAK2*
NRF2-mediated Oxidative Stress Response	duodenum (Darkgreen)	2.78	*NQO1,DNAJB1,DNAJA1*
	liver (Yellow)	4.15	*FOS,UBB,JUN,DNAJB4,GSTA4,STIP1,DNAJA4,HSPB8,DNAJA2,DNAJA1,MAFF,DNAJB5*
Role of RIG1-like Receptors in Antiviral Innate Immunity	duodenum (Darkred)	1.32	*CASP10*
	liver (Yellow)	1.50	*NFKBIE,NFKB2,NFKB1*
Unfolded protein response	duodenum (Darkgreen)	2.7	*HSPH1,HSPA2*
	liver (Yellow)	3.66	*HSPA4,INSIG1,HSPH1,PPP1R15A,DNAJA2,HSPA2*
Protein Ubiquitination Pathway	duodenum (Darkred)	1.48	*HSPE1,ANAPC10*
	duodenum (Darkgreen)	4.89	*HSPH1,DNAJB1,DNAJA1,HSPA2,HSPA4L*
	liver (Yellow)	5.40	*UBB,CRYAB,DNAJB4,HSPH1,HSPB8,THOP1,HSPD1,DNAJA1,HSPA2,HSPA4,HSP90AB1,UBE2B,NEDD4L,DNAJB5,HSPA4L,BIRC2*

### Identification of candidate genes associated with FE in liver and duodenum tissues

Intra-module analyses revealed interesting tissue-specific candidate genes (i.e. detected only in one of the two analysed tissues), as well as 12 candidate genes that were commonly identified in liver and duodenum transcriptomes (Table 3). Supplementary Table S4 shows the whole list of genes included in each of the four gene co-expression modules associated to FE. Additionally, a list of genes contained in these co-expression modules that has been previously reported as associated with FE phenotypes in pigs or other livestock species is provided in Supplementary Table S6.

**Table 3:** List of genes in co-expression modules associated to feed efficiency identified in both liver and duodenum tissues.

Ensembl ID	Symbol	Chr	Start (bp)	End (bp)	Biotype
ENSSSCG00000002274	*HSPA2*	7	94987618	94990126	protein_coding
ENSSSCG00000002535	*HSP90AA1*	7	129758772	129761205	protein_coding
ENSSSCG00000009077	*HSPA4L*	8	103901031	103951701	protein_coding
ENSSSCG00000009334	*HSPH1*	11	7585525	7661067	protein_coding
ENSSSCG00000009636		14	8076243	8084520	protein_coding
ENSSSCG00000010686	*BAG3*	14	141034618	141058597	protein_coding
ENSSSCG00000011000	*DNAJA1*	10	37686862	37699607	protein_coding
ENSSSCG00000013554	*TRIP10*	2	72685806	72695425	protein_coding
ENSSSCG00000013784	*DNAJB1*	2	65177577	65180388	protein_coding
ENSSSCG00000014399	*ARHGAP26*	2	150495712	150918983	protein_coding
ENSSSCG00000021620	*STIP1*	2	6978383	7040372	protein_coding
ENSSSCG00000030182	*DEDD2*	6	45629733	45646518	protein_coding

Interestingly, approximately 20% (90 out of 440) of genes identified in the liver Yellow module were reported as associated with FE in pigs and/or cattle^13,23^. For example, Serum/Glucocorticoid Regulated Kinase 1 (*SGK1*) gene had been previously identified as linked to pig eating behaviour through a GWAS^28^. It should be noted that differences in eating behaviour between extreme RFI pigs have been described in previous studies^29^. *SGK1* was also reported as differentially expressed between animals divergently selected for RFI in two broiler chicken lines^14,15^. This gene encodes a serine/threonine protein kinase that plays an important role in cellular stress response and in the regulation of a wide variety of processes including ion channel transport and mTOR signaling pathways which was over-represented in our study. Other members of the liver Yellow module such as Growth Arrest and DNA Damage Inducible Gamma (*GADD45G*) and Nuclear Factor Kappa B Subunit 1 (*NFKB1*) had been reported as DE in the liver of RFI-divergent beef cattle^8^ and chicken^30^.

Focusing on duodenum co-expression modules associated to FE, ~32% (12 out of 37) of gene members of the Darkgreen module corresponded to genes already reported as candidate genes for efficiency-related traits in other species (Supplementary Table S6). Kern *et al*.^31^ suggested the ruminal expression of NAD(P)H Quinone Dehydrogenase 1 *(NQO1)* as indicator of FE in beef. Moreover, genes such as SIN3 Transcription Regulator Family Member A *(SIN3A)*, CUB And Sushi Multiple Domains 2 *(CSMD2)*, Leukocyte Cell Derived Chemotaxin 2 *(LECT2)* and Cell Division Cycle 37 Like 1 *(CDC37L1*) had been described as associated with FE traits in beef^9,12^ and poultry^30,32^. It is also worth highlighting the Interleukin 22 *(IL22)* gene that encodes for a cytokine relevant for intestinal barrier maintenance and functioning, implicated in tissue regenerative processes in gut and skin^33^. Transcription factors and candidate genes involved in lipid metabolism processes such as the Peroxisome Proliferator Activated Receptor Alpha *(PPARA)*, Acyl-CoA Thioesterase 4 *(ACOT4)* and Sirtuin 4 *(SIRT4)* were also identified in the duodenum Darkred module. The *ACOT4* and *PPARA* genes have been also reported as candidate genes for efficiency-related traits in chicken and pigs^15,23,34^.

### Common genes identified in both liver and duodenum gene modules

As stated above, 12 genes (out of 636 genes) were commonly identified as members of co-expressed modules associated to FE in both liver and duodenum tissues (Table 3). According to the current version of the pig genome annotation (*Sscrofa10.2*), a detailed examination of the 12 common genes associated with FE reveals that three of them, ENSSSCG00000009636, ENSSSCG00000002535 and ENSSSCG00000013554, encoding for uncharacterized proteins. The orthologues of two of those genes revealed a high homology with *HSP90AA1* and *TRIP10* genes, respectively (Table 3). Interestingly, 50% of the remaining ten genes encode for Heat Shock Proteins (HSPs): *HSPA2, HSPA4L, HSPH1, DNAJA1* and *DNAJB1*. HSPs are highly conserved proteins present in all organisms, being critical regulators in cellular stress response^35^. Heat stress reduces feed intake and affects both intestinal integrity and barrier function^36^. In pigs, heat stress increases mRNA expression of HSPs and reduces ileal abundance of several metabolic enzymes suggesting metabolic modifications^36^. A relationship between heat production and RFI in pigs has been proposed. Pigs belonging to the high RFI (low FE) line were energetically less efficient because of their greater heat production related to physical activity and basal metabolic rate^37^. In our study, a positive correlation was observed between the eigengene of modules carrying these genes (liver-Yellow and duodenum-Darkgreen) and RFI and FCR traits, suggesting that animals with higher expression of these genes are less efficient. In agreement with the WGCNA analysis, *HSPH1* was up-regulated in the liver of high RFI (low FE) compared to low RFI (high FE) group as well as other members of this gene family such as *HSPB8, CRYAB* and *HSPH1* (Supplementary Table 1).

In addition, these proteins are part of the common overrepresented pathways Aldosterone Signaling in Epithelial Cells, protein ubiquitination pathway and NRF2-mediated Oxidative Stress. Aldosterone is the main mineralocorticoid hormone synthetized in the adrenal gland playing a major role in the control of arterial blood pressure and extracellular volume homeostasis (reviewed in^38^). Remarkably, aldosterone has been related with an increase of feed intake and weight gain^39,40^. Aldosterone binds to the mineralocorticoid receptor (MR) which is expressed in different tissues exerting numerous functions (reviewed in^38^). Binding of HSP90 to MR is required for ligand binding activity^41^. In addition, aldosterone and MR activation are directly associated with the induction of oxidative stress (reviewed in^38^). In previous works, the decrease in reactive oxygen species (ROS) production and oxidative stress have been related to low RFI animals^23,42^. Furthermore, interconnected networks related to oxidative stress and immunity have been previously described as associated to FE^23^, supporting the involvement of immune system activation in the release of highly ROS^23^. In our study, pathways related to protein metabolism, immune response, and cellular defence mechanisms to maintain intracellular redox homeostasis were predicted to be up-regulated in the L_FE_ group. This result suggests that less efficient animals have a high protein turnover, activated inflammatory processes and high oxidative protein damage.

**Figure 2:**
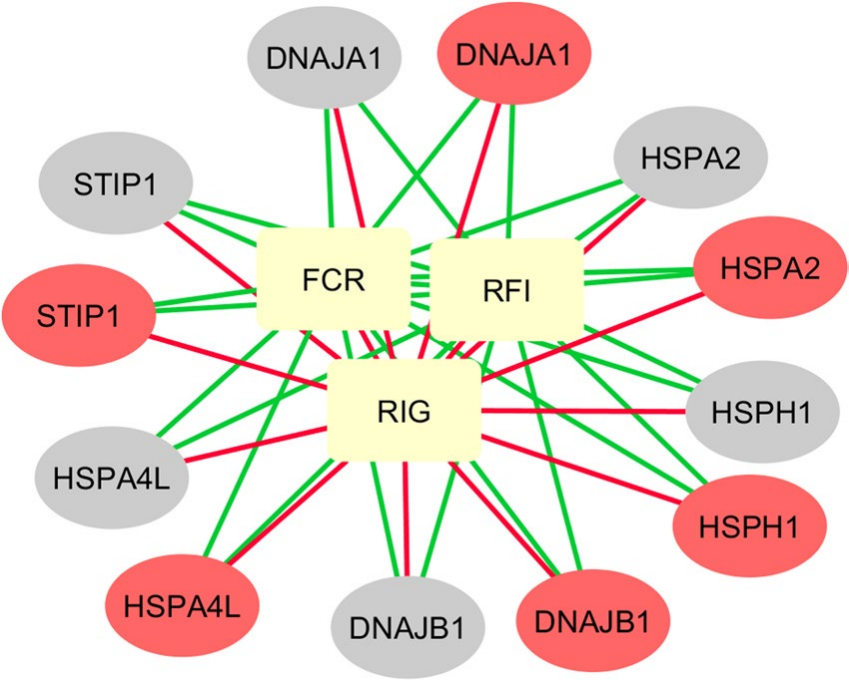
Correlation networks among expression levels of *STIP1, HSPA2, HSPA4L, HSPH1, DNAJA1* and *DNAJB1* genes in liver and duodenum, and their relationships with residual feed intake (RFI), residual intake and body weight gain (RIG), and feed conversion ratio (FCR). Node colour distinguish among tissue transcriptomic (red = liver; grey = duodenum) and phenotypic (yellow) information. Edge colour represents positive (green) and negative (red) correlation coefficients.

Interestingly, recent studies in beef steers^43,44^ have identified genes encoding for heat shock proteins and *DNAJ*, as well as those involved in the aldosterone pathway, as DE in the spleen and small intestine transcriptomes of animals phenotypically divergent for body weight gain and feed intake, supporting the implication of those genes and pathway in FE traits. Specifically, the HSP *HSPH1* and *DNAJA1*, jointly with the Stress Induced Phosphoprotein *(STIP1)* gene, were consistently identified in these studies. *STIP1* was also a differentially abundant protein between thermal neutral and heat-stressed pigs^36^. *STIP1* is an adaptor protein that coordinates the functions of *HSP70* and *HSP90* by stimulation of ATPase activity of *HSP70* and inhibition of ATPase activity of *HSP90*^45^. According to the String database (http://string-db.org/)^46^, experimental data confirmed a functional interaction among *STIP1*, *HSPA2*, *HSPA4L*, *HSPH1* and *DNAJA1*. In our study, strong and positive correlation ranging from 0.67 to 0.99 among the expression level of these genes was observed in both duodenum and liver tissues (Supplementary Figure 1). Opposite correlation patterns between individual gene expression and RFI and RIG phenotypic variation was observed (Fig. 2), consistently with aforementioned associations at module and phenotypic level. To detect potential candidate regulators among the 12 common genes, we performed an *in silico* identification of enriched TF-binding motifs and TFs with the iRegulon Cytoscape plugin. Different enriched motifs were detected, for example, Heat Shock Transcription Factor 1 (*HSF1*) was predicted as regulator of six of the 12 common genes: *HSP90AA1, STIP1, DNAJA1, HSPH1, DEDD2, DNAJB1* (Supplementary Table S7). All these observations suggested a coordinated interaction pattern and/or regulatory cascade involving HSPs in both liver and duodenum tissues that may have a role in the transcriptional regulation of FE in pigs.

### Identification of putative master transcriptional factors in the co-expression modules

The *in silico* identification of enriched TF-binding motifs provided different TFs that might regulate candidate genes in a tissue-specific manner (see Supplementary Table S7). Potential regulators of FE co-expression modules in duodenum included TF such as *Tumor Protein P53 (TP53), Distal-Less Homeobox 1 (DLX1), TATA-Box Binding Protein Associated Factor 1 (TAF1), Activating Transcription Factor 5 (ATF5), Nuclear Respiratory Factor 2 Alpha Subunit (GABPA)* and *HMG-Box Transcription Factor 1 (HBP1)*. In liver, however, a low number of putative TF were identified despite the high number of genes gathered by the liver Yellow module; potential TF identified in liver included *Heat Shock Transcription Factor 1 (HSF1)*, *ATP-Dependent Helicase CHD1 (CHD1)*, *SIN3 Transcription Regulator Family Member A (SIN3)* and *RNA Polymerase II Subunit A (POLR2A)*. A comparison between TFs identified in liver and duodenum revealed *POLR2A* as the only common putative regulator. This result together with the low overlapping between tissues at candidate gene and functional levels suggest different mechanisms modulating the transcriptional expression of genes related with pigs FE in liver and duodenum tissues.

### Common genes and pathways identified in DE and WGCNA analyses

In this study we used a combination of DE and WGCNA analyses; 12 genes identified as DE in liver (7 genes) and duodenum (5 genes) were also included in co-expression modules associated with the analysed traits (Table 4). Three of these genes encode for the HSPs *HSPB8, CRYAB* and *HSPH1*, and were up-regulated in the L_FE_ (inefficient) group (Supplementary Table 1), thus agreeing with the previously mentioned association between HSP gene expression and phenotype variation in RFI and FCR (Fig. 2). *HSPB8* had been previously reported as associated with RFI in a GWAS^47^, as well as DE in the liver of Nelore steers with divergent RFI phenotypes^48^. *CRYAB* gene acts as a molecular chaperone and is a member of the small heat shock protein family. Previous studies in several species also described associations of this gene with FE. *CRYAB* was found up-regulated in broilers with low FE^26^, and DE between individuals with divergent FE phenotypes in cattle (jejunum) and pigs (adipose tissue)^23,43^. Among the other common genes, *ATF3* and *MYOM1* have also been previously reported as candidate genes for FE traits^48–50^*. ATF3* encodes a member of the mammalian activation transcription factor/cAMP responsive element-binding (CREB) protein, and is involved in cellular stress response. Interestingly, a member of this family of TF (ATF5) was *in silico* predicted among the top putative regulators by iRegulon (Supplementary Table 8). As previously observed across tissues, a better overlap was observed at functional level between results from DE and WGCNA analyses: 15 biological pathways were identified with both approaches (Supplementary Table 9). These results suggest the usefulness of pathways-centred analysis to carry out intervention studies (*e.g*. nutritional) targeting these specific pathways to improve FE in pigs.

**Table 4:** List of genes associated to feed efficiency commonly identified in a differential expression (DE) and a co-expression network based analyses.

Tissue (module)	#Genes	Ensembl ID	Symbol	DE Tissue
liver (Yellow)	12	ENSSSCG00000003693	*MYOM1*	duodenum
		ENSSSCG00000009844	*HSPB8*	duodenum
		ENSSSCG00000015025	*CRYAB*	duodenum
		ENSSSCG00000021873	*CRABP1*	duodenum
		ENSSSCG00000027609	*GC*	duodenum
		ENSSSCG00000000181	*RND1*	liver
		ENSSSCG00000007554	*ZFAND2A*	liver
		ENSSSCG00000009334	*HSPH1*	liver
		ENSSSCG00000015595	*ATF3*	liver
		ENSSSCG00000023589		liver
		ENSSSCG00000024048		liver
		ENSSSCG00000030165	*MAFF*	liver
Duodenum (Darkgreen)	1	ENSSSCG00000009334	*HSPH1*	liver

## Conclusions

This study provides a list of candidate genes and pathways associated with FE in pigs. A differential expression analysis between animals with divergent RFI revealed DE genes in liver and duodenum tissues, whereas a more integrative network-based approach allowed identifying four modules of co-expressed genes that were associated to FE traits. Despite the low number of common genes identified in both liver and duodenum modules, an interesting overlapping between tissues was observed at functional (pathways) level, suggesting that gene members of the same biological pathways are differentially regulated in liver and duodenum tissues. Among biological pathways overrepresented in both liver and duodenum, those related to response to oxidative stress, inflammation and immune response seemed consistently involved in FE related traits. These results provide novel insights into the molecular mechanisms controlling FE in pigs, support the usefulness of pathways-centred analysis, and reveal the convenience of using integrative approaches combining omics and phenotypic information for disentangling the regulatory role of genes in a complex system.

## Material and Methods

### Animals, phenotypes and samples

Animal material and data come from a trial focusing on pigs FE under different feeding strategies that was carried out in the framework of the European ECO-FCE project (A whole-systems approach to optimise feed efficiency and reducing the ecological footprint of monogastrics).The trial was conducted between December 2014 and March 2015 at IRTA Pig Experimental Farm (Monells, Spain) with up to 244 individuals (121 females and 123 males) selected from 25 litters from Hermitage hybrid females inseminated with 4 controlled Hermitage Maxgro boars. Animals were involved in the trial from around 25 kg of body weight (BW) until around 106 kg BW. On average the animals were 69 (68–70) and 153 (152–154) days old at the beginning and at the end of the trial. Pigs were weighed individually at the start of the trial, every three weeks (weeks 3, 6, 9 and 12), and at the end of the study (weeks 14–15). Average daily weight gain (ADG) was calculated. Backfat thickness (BFT) was also measured every 3 weeks. Individual feed intake (FI) was recorded by the electronic feeders located in each pen (IVO-station feeder; INSENTEC^®^). All animals were offered the same type of feed but following 4 different feeding protocols that had no significant effects on production traits. Animal care and procedures were performed according Spanish and European regulations about the protection of animals used in experimentation, following national and institutional guidelines for the Good Experimental Practices and approved by the Ethical Committee of the Institut de Recerca i Tecnologia Agroalimentàries (IRTA).

Different measures of individual FE during the controlled fattening period were computed, establishing the RFI as the criterion to classify animals regarding FE. RFI was computed as deviation of actual amount of feed consumed during the control period (69–153 days) from the expected consumption for covering main biological functions such as maintenance, growth and fat deposition. This way, RFI corresponds to the residual of the following model:

1 $$DF{I}_{ij}={S}_{j}+{\beta }_{MW(j)}M{W}_{i}+{\beta }_{ADG(j)}AD{G}_{i}+{\beta }_{BFT(j)}BF{T}_{i}+RF{I}_{ij}$$

where *DFI*_*ij*_ is the daily feed intake from 69 to 153 days of age of the *i*^*th*^ individual of sex *j* (male or female); *S*_*j*_ is the effect of the *j*^*th*^ sex level (j = 1, 2) on DFI; *MW*_*i*_*, ADG*_*i*_ and *BFT*_i_ are, respectively, the metabolic weight at mid-point of the trial (~110.5 days of age), the average daily gain across the control period, and the backfat thickness at the end of the control period of the *i*^*th*^ individual; *β*_*MW*(*j*)_, *β*_*ADG*(*j*)_ and *β*_*BFT*(*j*)_, are the partial regressions coefficients, nested to sex, of DFI on MW, ADG and BFT; and finally *RFI*_*ij*_ is the residual feed intake of individual *i*, i.e. the residual of the regression model. Feeding protocol was excluded from the analysis model after checking the absence of significant effects on either DFI, ADG and BFT.

Additionally, other measures of FE such as FCR (kg of FI/kg of ADG) and RIG were computed. The RIG phenotype, that consider the residual weight gain in addition to RFI, were calculated according to Berry and Crowley^7^ applying the following formula:

2 $$RI{G}_{i}=[(R{G}_{i}/S{D}_{RG})-(RF{I}_{i}/S{D}_{RFI})]$$

where *RG*_*i*_ is the residual body weight gain of individual *i*, obtained as the residual of a regression model of ADG on weight, feed intake and fat deposition (i.e. $AD{G}_{i}={S}_{j}+{\beta }_{MW(j)}M{W}_{i}+{\beta }_{ADG(j)}DF{I}_{i}+{\beta }_{BFT(j)}BF{T}_{i}+R{G}_{i}$); SD_RG_ and SD_RFI_ correspond to the standard deviations of RG and RFI in the analysed sample.

At the end of the trial, individual FE was assessed by RFI values and 40 females were chosen among the most extreme animals: 20 with low RFI (group of high FE) and 20 with high RFI (group of low FE). Despite no significant differences across feeding protocols were observed, the selection of animals for RNA-Seq experiment considered a balanced design for dietary treatments (i.e. included the 5 best and 5 worse feed convertor females within each treatment). These animals were slaughtered 10–12 days after the end of the FE-testing period, at ~163 days of age, at IRTA’s experimental slaughterhouse in Monells (Spain), where the collection of biological samples took place just after sacrifice. Samples of approximately 1 mg were taken from the most relevant tissues (liver, duodenum, back-fat and hypothalamus); all samples were immediately submerged in RNA-later, and stored at −80 °C after 24 h. In the present study, the transcriptome of duodenum epithelium and liver tissues belonging to these females was explored, in association with their phenotypes for RFI, FCR and RIG.

### RNA isolation, library preparation and sequencing

Total RNA of 40 females was isolated from liver and duodenum using the RiboPure^TM^ Isolation of High Quality Total RNA (Ambion^®^; Austin, TX) following the manufacturer’s recommendations. RNA was quantified using the NanoDrop ND-1000 spectrophotometer (NanoDrop products; Wilmington, USA) and checked for purity and integrity with a Bioanalyzer-2100 (Agilent Technologies, Inc.; Santa Clara CA, USA). After quality control, three samples were excluded due to low RNA quality. Each library was being paired-end sequenced (2 × 75 bp) using the Illumina TruSeq SBS Kit v3-HS, on a Illumina HiSeq. 2000 platform at *Centro Nacional de Análisis Genómico* (CNAG-CRG; Barcelona, Spain).

### Mapping, assembling and annotation of reads

Quality control for the raw reads sequenced was analysed with FASTQC (Babraham Bioinformatics; http://www.bioinformatics.babraham.ac.uk/projects/fastqc/). Reads were mapped against the reference pig genome (*Sscrofa10.2*) and the annotation database Ensembl Genes 86 using the open-source software STAR 2.5.2a^51^. Mapping quality evaluation and descriptive statistics were generated with Qualimap v.2.2^52^. The resulting bam files containing the aligned sequences were subsequently merged with Samtools^53^. The total number of genome-mapped reads to gene was quantified using HTSeq. 0.6.1p2^54^ with the same GTF file used for the alignment step.

### Differential expression

The differential expression analysis contrasting the extreme samples was performed using the common dispersion and the ‘glmFit’ function of the edgeR package^55^. The fitted model includes feeding protocol (4 levels) as a fix effect. Supplementary table S10 resume the full list of the used genes as well as their read counts data. Genes were considered as DE when the rate of change between groups reached |Fold Change (FC)| > 1.5 and differences were significant after adjusting for multiple testing (FDR < 0.05).

### Validation of differentially-expressed genes by RT-qPCR

In order to evaluate the repeatability and reproducibility of gene expression data obtained by RNA-Seq, a quantitative real-time PCR (qPCR) assay using SYBR Green chemistry (SYBR^TM^ Select Master Mix, Applied Biosystems) and the comparative Ct method^56^ was performed. The isolated RNA of individual samples for liver and gut tissues was reverse-transcribed into cDNA using the PrimeScript RT Reagent Kit (TAKARA) in a total volume of 20 μl containing 1 μg of total RNA, following the manufacturer’s instructions. All primers were designed using PrimerExpress 2.0 software (Applied Biosystems) (Supplementary Table S11). The *ACTB* and *HPRT1* genes were used as endogenous controls^57^.

All assays were tested and PCR efficiencies were evaluated by performing standard curves with a four-fold dilutions series (1/4, 1/16, 1/64, 1/256, 1/1024) per triplicate of a pool of 6 cDNA samples in an Applied Biosystems 7500 Real-Time PCR System (Applied Biosystems, Inc.; Foster City, CA). A dissociation curve was drawn for each primer pair. A QuantStudio™ 12 K Flex Real-Time PCR System (Applied Biosystems, Inc.; Foster City, CA) was used for mRNA quantification. The reactions were carried out in a 96-well plate for the ABI PRISM® 7900HT instrument in a 20 μl volume containing 5 μl of cDNA sample diluted 1/20. For the QuantStudio™ 12 K Flex Real-Time PCR instrument, the reactions were carried out in a 384-well plate in 15 μl volume containing 3.75 μl of cDNA sample diluted 1/20. All primers were used at 300 nM. The thermal cycle was: 10 min at 95 °C, 40 cycles of 15 sec at 95 °C and 1 min at 60 °C. Each sample was analysed in duplicate. Data was analysed using the Thermo Fisher Cloud software (Applied Biosystems). The sample of lowest expression level was selected as calibrator. Correlation coefficients between RNA-seq and qPCR data (RQ) as well as group mean comparison test were computed with R.

### Weighted Gene Coexpression Network Analysis (WGCNA)

The RNA-Seq expression data corresponding to the complete list of expressed genes in each tissue (Supplementary Table S10) was normalized using the edgeR package^55^ and then adjusted for the fixed effects of feeding protocols (four levels) with the linear model procedure of R. To identify co-expressed and highly interconnected genes associated with RFI, FCR and RIG the WGCNA approach was implemented as follows:

1. The network construction as well as the identification of the modules of co-expressed genes was done separately for each tissue (liver and duodenum). The soft-thresholding power as function of the scale-free topology index was defined at six, which represent a power-law model in liver of R^2^ = 0.90 and R^2^ = 0.97 in duodenum.
2. In each tissue, Pearson’s correlation between the module eigengene and the phenotype information was estimated. The eigengene, is defined as the first principal component of a given module and can be considered a representative of the gene expression profiles in a module^20^. A module was chosen for downstream analysis if it presented module-trait relationship ≥ |0.25| in at least two of the three analysed traits (RFI, FCR and RIG), and p-value ≤ 0.05 in at least one of these traits.
3. In order to identify common genes associated with RFI, FCR and/or RIG in both tissues, a comparison between those genes reported in each tissue was performed.

### Identification of regulators, functional classification and pathway analyses

To detect potential regulators among the candidate genes, an *in silico* identification of enriched transcription factor (TF)-binding site motifs in the *cis*-regulatory elements of the candidate genes was done using the iRegulon v1.3 Cytoscape plugin^58^. Gene function classification and pathway analyses were performed using Ingenuity Pathways Analysis software (IPA, Ingenuity Systems; Redwood City, CA). The cut-off for considering a significant over-representation was established with a p-value ≤ 0.05 after Benjamini and Hochberg multiple-test correction^59^.

### Data availability

RNA-Seq data has been submitted to NCBI Bioproject under accession SUB2967615.

## Electronic supplementary material

Supplementary Material

Suplementary Table S1

Suplementary Table S2

Suplementary Table S3

Suplementary Table S4

Suplementary Table S5

Suplementary Table S6

Suplementary Table S7

Suplementary Table S8

Suplementary Table S9

Suplementary Table S10
