# Supplementary material for: Integrative approach using liver and duodenum RNA-Seq data identifies candidate genes and pathways associated with feed efficiency in pigs
Source: Sci Rep. 2018 Jan 11;8:558. doi: 10.1038/s41598-017-19072-5 (PMC5764994; doi:10.1038/s41598-017-19072-5)

Integrative approach using liver and duodenum RNASeq data identifies candidate genes and pathways associated with feed efficiency in pigs

Yuliaxis Ramayo-Caldas[1*], Maria Ballester[1], Juan Pablo Sánchez[1], Olga González-Rodríguez[1], Manuel Revilla[2,3], Henry Reyer[4], Klaus Wimmers[4,5], David Torrallardona[6], Raquel Quintanilla[1*]

[1]Animal Breeding and Genetics Program, Institute for Research and Technology in Food and Agriculture (IRTA), Torre Marimon, Caldes de Montbui, 08140, Spain; [2]Animal Genomics Department, Centre for Research in Agricultural Genomics (CRAG), Campus UAB, Bellaterra, 08193, Spain; [3]Departament de Ciència Animal i dels Aliments, Universitat Autònoma de Barcelona (UAB), 08193, Bellaterra, Spain; [4]Institute of Genome Biology, Leibniz Institute for Farm Animal Biology (FBN), Wilhelm-Stahl-Allee 2, Dummerstorf, 18196, Germany; [5]Faculty of Agricultural and Environmental Sciences, University Rostock, Rostock, 18059, Germany [6]Animal Nutrition Program, IRTA, Mas de Bover, Constantí, 43120, Spain.

*Corresponding author: yuliaxis.ramayo@irta.cat and raquel.quintanilla@irta.cat

Supplementary Information

Supplementary Table S1. Description of the differentially expressed genes detected in liver and duodenum between L_{FE} and H_{FE} groups of pigs

Supplementary Table S2. Genes reported as differentially expressed in pig lines divergent selected for RFI.

Supplementary Table S3. Pathways differentially-modulated between low efficient (L_{FE}) and high efficient (H_{FE}) groups of pigs in liver and duodenum.

Supplementary Table S4. List of gene module membership in liver and duodenum tissue

Supplementary Table S5. Over-represented pathways in genes gathered by FE associated co-expression modules identified in liver and duodenum.

Supplementary Table S6. Genes grouped in FE associated co-expression modules in liver and duodenum that had been reported as associated with feed efficiency in pigs and/or other species.

Supplementary Table S7. Putative transcription factors (TF) that may be regulating candidate genes (identified by *in silico* detection of enriched TF binding motifs).

Supplementary Table S8. Putative transcription factors (TF) that may be regulating the 12 common candidate genes (identified by *in silico* detection of enriched TF binding motifs).

Supplementary Table S9. Canonical pathways commonly identified by the DE and WGNCA analysis.

Supplementary Table S10. Description and read counts data of the genes employed in the analysis.

Supplementary Table S11. Primers used for gene expression quantification by qPCR.

Supplementary Table S11. Primers used for gene expression quantification by qPCR.

Primer Name	Sequence[a]
ATF3-RT-Fw	5'- TCA GTC ACG AAA GCC GAG GTA -3'
ATF3-RT-Rv	5'- GGC GGC GGC AAT CTT ATT -3'
CRYAB-RT-Fw	5'- AGG AAC TCA AGG TCA AGG TGT TG -3'
CRYAB-RT-Rv	5'- ATG TTC ATC CTG GCG CTC TT -3'
HSPB8-RT-Fw	5'- GGC AAA CAC GAG GAG AAG CA -3'
HSPB8-RT-Rv	5'- CAC CTC TGC AGG AAG CTG AAT T -3'
HSPH1-RT-Fw	5'- TGG TGG AGT TGG AAT AAA GGT CAT -3'
HSPH1-RT-Rv	5'- TGG CTG TTA TCT GCT CCA CAC T -3'
HPRT1-RT-Fw	5'- TCA TTA TGC CGA GGA TTT GGA -3'
HPRT1-RT-Rv	5'- CTC TTT CAT CAC ATC TCG AGC AA -3'
ACTB-RT-Fw	5'- CAA GGA CCT CTA CGC CAA CAC -3'
ACTB-RT-Rv	5'- TGG AGG CGC GAT GAT CTT -3'

Supplementary Figure S1. Co-expression patterns among *STIP1, HSPA2, HSPA4L, HSPH1, DNAJA1* and *DNAJB1* within and across liver and duodenum tissues.

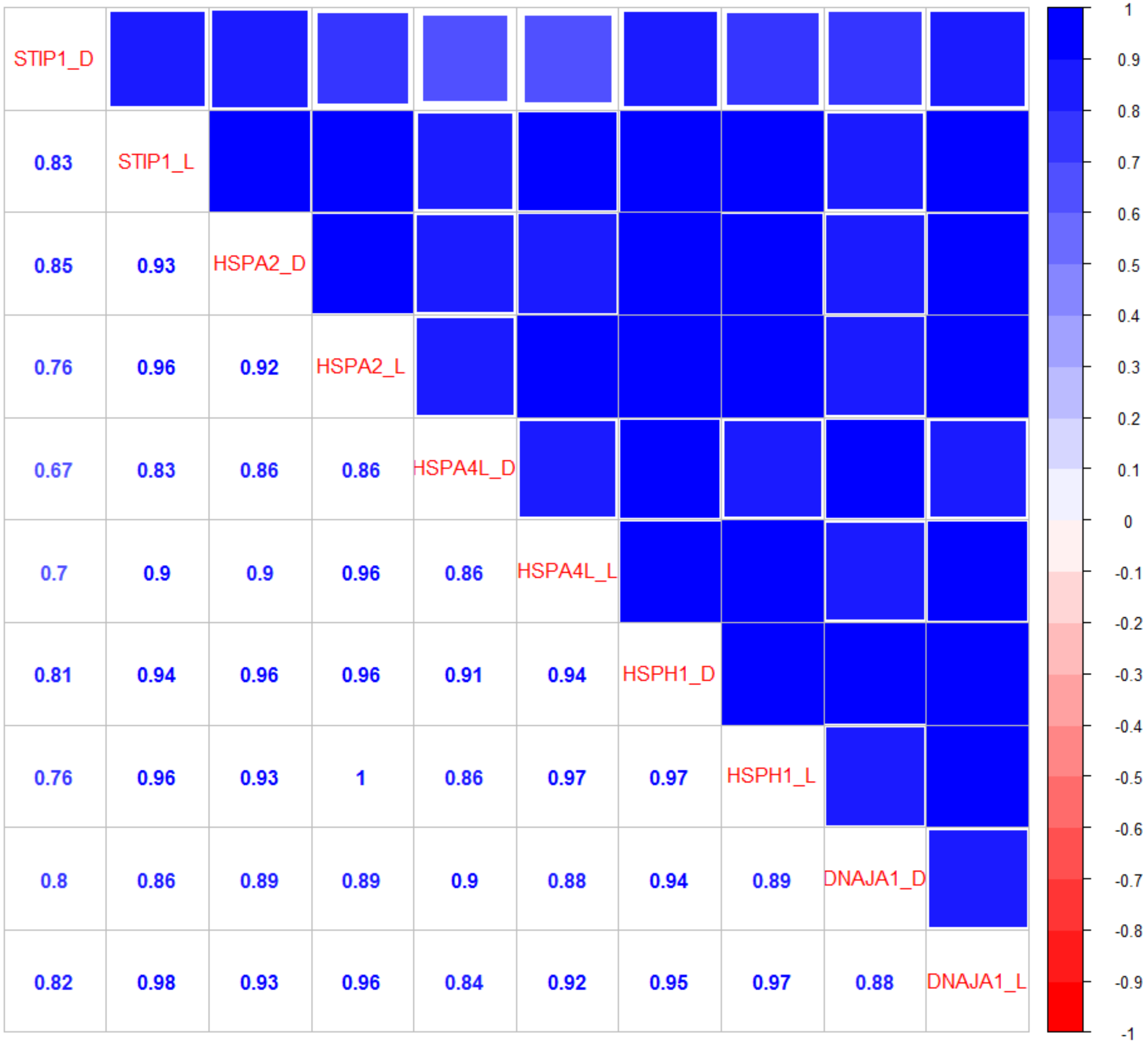

Supplement: Supplementary file 1 — Supplementary Material [file 41598_2017_19072_MOESM1_ESM.pdf]
